# Symptoms of Selective Mutism in Middle Childhood: Psychopathological and Temperament Correlates in Non-clinical and Clinically Referred 6- to 12-year-old Children

**DOI:** 10.1007/s10578-023-01512-1

**Published:** 2023-02-28

**Authors:** Peter Muris, Leonie Büttgens, Manouk Koolen, Cynthia Manniën, Noëlle Scholtes, Wilma van Dooren-Theunissen

**Affiliations:** 1 Department of Clinical Psychological Science, Faculty of Psychology and Neuroscience, Maastricht University, P.O. Box 616, 6200 MD Maastricht, The Netherlands; 2 Stellenbosch University, Stellenbosch, South Africa; 3 Youz-Parnassia Group, Oosterbeek, The Netherlands

**Keywords:** Selective mutism, Social anxiety, Behavioral inhibition, Autism spectrum problems, Clinical and non-clinical children

## Abstract

The purpose of this study was to study psychopathological and temperamental correlates of selective mutism (SM) (symptoms) in a mixed sample of non-clinical (*n* = 127) and clinically referred (*n* = 42, of whom 25 displayed the selective non-speaking behavior that is prototypical for SM) 6- to 12-year-old children. Parents completed questionnaires to measure their child’s symptom levels of selective mutism, social anxiety, autism spectrum disorder, and the temperament trait of behavioral inhibition. The results first and foremost showed that SM symptoms were clearly linked to social anxiety and an anxiety-prone temperament (behavioral inhibition), but findings also suggested that autism spectrum problems are involved in the selective non-speaking behavior of children. While the latter result should be interpreted with caution given the methodological shortcomings of this study, findings align well with the notion that SM is a heterogeneous psychiatric condition and that clinical assessment and treatment need to take this diversity into account.

## Introduction

Selective mutism (SM) is a psychiatric condition in which a child consistently fails to speak in specific social situations (e.g., school) despite having mastered language skills and speaking normal in other, more familiar situations (e.g., at home). To establish the diagnosis in terms of the *Diagnostic and Statistical Manual of Mental Disorders* (DSM), the nonspeaking behaviour should be persistent (i.e., have a duration of longer than 1 month) and hinder the child significantly in its functioning at school and during social interactions with others. Further, the failure to speak is not attributable to a lack of knowledge of, or comfort with, the spoken language, and does not exclusively occur during the course of autism spectrum disorder, schizophrenia or another psychotic disorder [1]. Across various clinical and non-clinical samples, between 0.3% and 1.9% of the children have been found to meet the full classification criteria of SM [2], but it is good to be aware that just like other mental health problems selective non-speaking behaviour is situated on a continuum, implying that there are also children displaying less severe symptoms of the disorder. In these cases, the lack of speech has not generalized across multiple situations, and speaking is less impaired in terms of quantity (e.g., the child uses less words in specific situations) and/or quality (e.g., the child’s does speak but with a whispering voice) [3].

In psychiatric classification systems, SM is currently considered as an anxiety disorder [4, 5]. The evidence to support this notion comes from three lines of research. First, a large number of studies have demonstrated that anxiety is a prominent symptom of children with SM. For instance, Vogel et al. [6] noted that fear and anxiety are common in children with this disturbance and a meta-analysis by Driessen et al. [7] even showed that the vast majority of children with SM (i.e., 80%) can also be diagnosed with a comorbid anxiety disorder. In particular, symptoms of social anxiety disorder appear to be highly prevalent in children with SM [8, 9]. The second research line has indicated that similar risk and vulnerability factors seem to be involved in the aetiology of SM as in other anxiety disorders. A good example is behavioural inhibition, a temperament trait characterized by a strong withdrawal tendency in response to novelty [10], which is generally regarded as a marker of anxiety proneness and a predictor of anxiety pathology [11] and social anxiety in particular [12], but also appears to be a prominent feature of children with SM [13, 14]. The third and final research line is concerned with treatment: just like in other anxiety disorders, evidence indicates that cognitive-behavioural interventions are particularly effective for treating children with SM [15─^17^].

In spite of all the support for the notion that SM is an anxiety disorder, there are also echoes in the psychological literature suggesting that the condition is more heterogeneous in nature. For example, empirical clinical profiles studies have indicated that besides anxiety other problems often occur in children with SM as well. These include oppositionality, developmental and language delays, communication difficulties, and other social problems [18─^20^]. Given this evidence, Kearney and Rede [21] concluded that SM is a complex and multifaceted disorder that possibly can better be conceptualized as a neurodevelopmental problem. In line with this conclusion, there are indications that SM is associated with autism spectrum disorder (ASD). Although it should be noted that relatively few studies have explored this issue, probably as a result of the rather ambivalent exclusion criterion as adopted in the DSM, it has been found that clinically referred children with SM often have a comorbid diagnosis of ASD [22, 23] and display elevated scores on measures of autistic traits [24, 25]. Admittedly, there is also research showing that children with SM do not exhibit heightened scores on a quantitative scale of autistic traits [26], but it is important to note that this study excluded children with SM who were high on the autism spectrum. In the reality of common clinical practice, it seems to be the case that SM and ASD are not mutually exclusive conditions and that the (selective) non-speaking behaviour of children is not only based on (social) anxiety but also may be fuelled by ASD symptomatology [27].

In our previous investigation of this hypothesis, we explored the relative contributions of social anxiety, anxiety proneness (i.e., the temperament trait of behavioral inhibition), and ASD symptoms to symptoms of SM [28]. Constructs were measured by means of a set of parent-report questionnaires that were completed by the mothers and/or fathers of 3- to 6-year-old non-clinical children (*N* = 172). The results showed that there were positive and statistically significant correlations between SM and social anxiety, behavioral inhibition, and ASD symptoms. Regression analyses revealed that (a) both social anxiety and ASD symptoms accounted for a significant and unique proportion of the variance in SM scores, and (b) that both of these variables no longer made a significant contribution once behavioral inhibition was added to the model. Apparently, this temperament trait covered children’s fearful responding in social situations as well as the interaction and communication difficulties associated with the autism spectrum. It was concluded that while the involvement of social anxiety and anxiety-proneness is indisputable in SM, autism-related problems are also to some extent implicated.

The present research employs a similar method as the earlier Muris et al. [28] study. Thus, a parent-report survey was used to assess the constructs of social anxiety, behavioral inhibition, ASD problems, and SM symptoms in children. The method deviated in two ways from our previous investigation. First, although SM is an early onset disorder that usually becomes manifest during the preschool years [1], it is clear that the selective non-speaking may continue into the middle childhood years (and even beyond [29]). The current study focused on this age group and included children aged 6 to 12 years. Second, besides a sample of non-clinical children (Sample 1), the present study also included clinically referred children with SM and children with other social-emotional psychopathology (Sample 2). In keeping with our previous study [28], we expected to find positive correlations between social anxiety, behavioral inhibition, and ASD symptoms on the one hand and symptoms of SM on the other hand. We also anticipated that social anxiety and ASD symptoms would each explain a unique and significant proportion of the variance in symptoms of SM, but that these variables would no longer make a significant contribution once the temperament trait of behavioral inhibition was added to the model [28]. Finally, in the clinically referred children with SM, we anticipated not only higher levels of social anxiety and behavioral inhibition but also elevated levels of ASD symptoms. The latter finding would of course provide a further indication for the role of this neurodevelopmental disorder in the selective non-speaking behavior of these young people.

## Method

### Participants and Procedure

Sample 1 consisted of 127 non-clinical children (62 girls and 65 boys; mean age = 8.69 years, *SD* = 1.82, range 6–12 years) who were recruited in four elementary schools in the southern part of the Netherlands and via a snowball sampling procedure among acquaintances of the researchers. In the schools, a flyer with the title “Kinderen die soms niet spreken” (“Children who sometimes do not speak”) was spread among the parents of children in school classes 3 to 8 and this flyer was also posted and shared on social media channels of people in the social network of the researchers. To participate in the study, parents should have (1) sufficient mastery of the Dutch language in order to be able to complete our survey, and (2) a child with an age between 6 and 12 years who had not been diagnosed with SM. Parents who indicated interest in participating were provided with an information letter along with a consent form. After they had provided consent, they received a weblink that guided them to the online survey. Initially, 159 parents responded positively by signing the informed consent form. However, the data of 32 parents had to be discarded because the survey was not (fully) completed (*n* = 26) or because the survey was filled out for a child who was younger or older than the intended age range (*n* = 6). In most cases, the biological mother completed the survey (*n* = 113, 89.0%); in other cases, both parents, the biological father, or a foster- or stepparent filled out the set of questionnaires. The participating parents were mainly born in The Netherlands (*n* = 114, 89.7%) and Belgium (*n* = 10, 7.9%), and only a few originated from other, non-Western countries (i.e., Syria, Surinam, and Indonesia; *n* = 3, 2.4%). In most families, the Dutch language (*n* = 112, 88.2%) or a derivative dialect (*n* = 13, 10.2%) was spoken at home; only in two families another foreign language (i.e., Arabic and Papiamento; *n* = 2, 1.6%) was dominant. In the vast majority of families, parents were either married or cohabitating (*n* = 107, 84.2%). The children of Sample 1 were not diagnosed with SM (which was an exclusion criterion) and currently not in clinical care, but 14 of them (11.0%) were reported to have been diagnosed with conditions such as attention-deficit (hyperactivity) disorder, learning disorder, developmental language disorder, or ASD.

Sample 2 was composed of 42 clinically referred children (19 boys and 23 girls; mean age = 8.17 years, *SD* = 2.62, range 6–12 years) who were clients of the Youz-Parnassia Group in Limburg. Parents were provided with an information letter and a consent form via mail or during an appointment at this outpatient treatment facility. In case they agreed to participate, they were either given a paper version of the survey or a link to the online platform on which they could fill out the set of questionnaires. Twenty-five of these children (11 boys and 14 girls) constituted the SM group as they were all displaying the prototypical selective non-speaking behavior associated with this disorder: all these children refrained from speaking to the psychologist during the intake assessment and did not speak in their class at school (which was confirmed during a formal contact with the teacher), while speaking normally to parents and siblings at home. The majority of them already had received the official diagnosis of SM in a previous clinical setting (60.0%), whereas in the other cases the selective non-speaking was the main reason for referral to the treatment facility and the clinical evaluation was still in progress. The other 17 children (8 boys and 9 girls) formed the clinical control group: they did not show the prototypical signs of SM but were referred to the facility because of social-emotional problems, with specific anxiety disorders (*n* = 6, i.e., separation anxiety disorder (*n* = 2), social anxiety disorder (*n* = 2), anxiety disorder not otherwise specified (*n* = 2)) and ASD (*n* = 11) being the primary diagnoses. These children went through the standard diagnostic procedure at the outpatient treatment facility, which included an unstructured interview with child and parents, standardized diagnostic instruments (such as the Autism Diagnostic Interview-Revised), and psychological assessment (e.g., intelligence test). In the total clinical sample, the survey was completed by the biological mother (*n* = 38, 90.5%) or biological father (*n* = 4, 9.5%) and most of the participating parents originated from The Netherlands (*n* = 37, 88.1%; other parents came from Belgium, Canada, The Philippines, and Turkey). The spoken language at home was again mostly Dutch or Dutch dialect (*n* = 39, 92.9%); in the other families (*n* = 3, 8.1%) English, Tagalog, or Turkish was spoken. Most families were complete (*n* = 33, 78.6%); in other cases, the parents had divorced (*n* = 9, 21.4%).

The current study was approved by the Ethics Review Committee of Psychology and Neuroscience (ERCPN) at Maastricht University under reference codes ERCPN 221_50_03_2020 and ERCPN 242_122_09_2021.

### Measures

The *Selective Mutism Questionnaire* (SMQ) [30] is a 17-item parent-report scale that can be used to measure the frequency of a child’s lack of speech across various settings: at school (e.g., “When appropriate, my child asks his or her teacher questions”), at home or with family (e.g., “When appropriate, my child talks to family members living at home when other people are present”), and in other social situations (e.g., “When appropriate, my child speaks with other children who s/he doesn’t know”). Items have to be rated on a 4-point scale with 0 = never, 1 = sometimes, 2 = often, and 3 = always. A total score can be obtained by summing scores across all items (range 0–51). Originally, a lower SMQ total score is indicative of a lower frequency of speaking and hence higher levels of SM, but for the purpose of this study SMQ scores were reversed so that higher scores reflect higher symptom levels. Bergman et al. [30] demonstrated that the SMQ has good internal consistency (with Cronbach’s alphas in the 0.80 to 0.90 range) and scores correlate positively with a variety of concurrent measures, which provides support for the validity of the scale. Similar positive psychometric properties of the SMQ have been documented in other countries [31, 32], including the Netherlands [33].

The social anxiety subscale of the *Children’s Anxiety Scale-Parent version* (CASP) [34] consists of 6 items that measure the frequency of children’s anxious behaviors and cognitions in social situations (e.g., “My child is afraid of talking in front of the class”, “My child worries that he/she will do something to look stupid in front of other people”, and “My child is scared to ask an adult for help (e.g., the school teacher)”. To enable comparison with earlier collected data of younger children, we used the items of the preschool version [35]. Parents are asked to indicate for each item how much the pertinent behavior or cognition applies to their child (0 = not at all true, 4 = very true). By summing the ratings across all items, a total score can be obtained (range 0–24) of which higher scores are indicative of higher levels of social anxiety. The CASP is in general a reliable and valid instrument for measuring anxiety in young children [35, 36] and there is also specific evidence for the validity of the social anxiety subscale [37]. There is no formal psychometric evaluation of the preschool CASP in children beyond the preschool age, but several studies (which made an attempt to study the phenomenon of childhood anxiety longitudinally) found that the measure, and the social anxiety in particular, is just as reliable (in terms of both internal consistency and test-retest stability) and valid (as indexed by correlations with other anxiety measures) in older children as when administered in preschool children [38, 39]. This finding is not surprising given that the CASP social anxiety scale essentially covers the same emotional and cognitive symptoms as its equivalent version for older children [36].

The short version of the *Behavioral Inhibition Questionnaire* (BIQ) [40, 41] was used to measure the temperament trait of behavioral inhibition, which is generally considered as an indicator of children’s anxiety proneness. The shortened BIQ contains 14 items (e.g., “My child is reluctant to approach a group of unfamiliar children to ask to join in”, “My child dislikes being the center of attention”) that parents have to rate on a 6-point Likert scale with 1 = almost never and 6 = almost always. Item scores can be summed to yield a total score, ranging from 14 to 84, with higher scores reflecting higher levels of behavioral inhibition. A psychometric evaluation of the BIQ by Vreeke et al. [42] has shown that the scale has excellent internal consistency (∝ = 0.92) and moderate test-retest stability correlations over periods of one year (*r* = .73) and two years (*r* = .65).

The parent version of the *Autism Spectrum Questionnaire* (ASQ) [43] is a 24-item scale for assessing autism spectrum symptoms in children aged 4 to 18 years. Items essentially cover the two key characteristics of ASD: impairments in social interaction and communication (e.g., “My child easily establishes contact with both boys and girls”, “My child actively seeks contact with peers”) and restrictive and repetitive behavioral interests (i.e., odd/deviant behaviors; e.g., “My child repeatedly talks and thinks about the same things”, “My child cannot deal well with changes”). Parents have to rate on a 5-point Likert scale how applicable the given statements are for their child, ranging from 1 (i.e., not at all) to 5 (i.e., very much). After recoding reversed items, a total score as well subscale scores for social interaction/communication problems and odd/deviant behaviors can be computed by summing the ratings on relevant items, with higher scores reflecting higher levels of ASD symptoms. Van der Ploeg and Scholte [43] reported good psychometric qualities for the scale, with excellent internal consistency (∝ = 0.94 for the total score and ∝ = 0.91 for both subscales) and test-retest stability (*r* = .91 for the the total score and ∝ = 0.91 for interaction/communication problems and ∝ = 0.84 for odd/deviant behaviors). Additionally, the ASQ demonstrated good validity: the scale was found to differentiate quite well between children with and without ASD (*n*’s being 254 and 1569; specificity = 91%, sensitivity = 85%, false positives = 9%, false negatives = 15%; Receiver Operator Characteristic, Area Under the Curve = 0.96) and correlated positively with another measure of autism spectrum problems (*r* = .73) [43].

### Statistical Analysis

Data were analyzed by means of the Statistical Package for the Social Sciences (SPSS, version 25). First, reliability coefficients (Cronbach’s alphas) were calculated for all scales and gender differences were explored by means of independent samples *t*-tests. Next, questionnaire scores were compared across the three groups (i.e., SM group, clinical control group, and non-clinical group) by means of analyses of variance (ANOVAs), which in the case of a significant effect were followed by Bonferroni-corrected post-hoc tests. Then, correlations were computed among various measures (for the total sample as well as for the separate samples of clinically referred and non-clinical children). We needed to be somewhat hesitant with computing correlations for the SM and clinical control groups separately, as a power analysis indicated that the sample size should be at least 40 for detecting the smallest correlation amongst these variables in our previous study [28] (i.e., r = .43, with α = 0.05 and ß = 0.20). Further, stepwise linear regression analyses were conducted in which the predictors social anxiety (CASP) and ASD symptoms (ASQ) were entered into the regression equation on step 1 – to explore their unique contributions to SM symptoms (SMQ; dependent variable), while behavioral inhibition (BIQ) was added to the model on step 2. Finally, crosstabs analyses were carried out to compare the proportions of children in the three groups for which elevated or even clinical scores on the ASQ were reported. The Fisher exact statistic was employed because of small numbers of children in some cells.

## Results

### Descriptive Statistics

Before discussing the main results of the study, some general findings need to be addressed. To begin with, all questionnaires were reliable in terms of internal consistency. That is, in all three groups (i.e., SM group, clinical control group, and non-clinical group), mostly sufficient to excellent reliability coefficients were found: more specifically, Cronbach’s alpha values were respectively 0.87, 0.92, and 0.94 for the SMQ, 0.60, 0.79, and 0.89 for the CASP, 0.89, 0.77, and 0.94 for the BIQ, 0.90, 0.81, and 0.93 for the total score of the ASQ, and between 0.86 and 0.91 for ASQ subscales. The relatively low Cronbach’s alpha of the CASP social anxiety scale in the SM group was considered as acceptable given that this scale only consisted of 6 items and all children in this group scored relatively high on this measure, which resulted in a low overall variance of item scores. Second, independent samples *t*-tests revealed only one statistically significant gender difference: for boys, parents reported higher scores on the ASQ and thus higher levels of ASD than for girls [means being 56.70, *SD* = 15.73 versus 51.44, *SD* = 15.06; *t*(167) = 2.22, *p* < .05]. Third and finally, a comparison of the three groups by means of ANOVAs (with Bonferroni-corrected post-hoc tests) revealed statistically significant effects for all questionnaires [all *F*(2,166)’s ≥ 12.53, *p*’s < 0.001]. As can be seen in Table 1, the SM group displayed significantly higher levels of selective mutism symptoms on the SMQ than the clinical control group (*p* < .001), which in turn scored higher than the non-clinical group (*p* < .05). Further, a similar pattern was noted for the BIQ: the SM group exhibited the highest levels of temperamental behavioral inhibition, followed by the clinical control group and the non-clinical group (with all between-group differences being significant at *p* < .01). On the CASP, children with SM were rated as showing significantly higher levels of social anxiety as compared to both the clinical control and the non-clinical groups (*p*’s < 0.001), which did not differ from each other. Finally, the SM group and the non-clinical control group did not show statistically different scores on the ASQ, but both clinical groups appeared to display significantly higher levels of autism spectrum symptoms as compared to the non-clinical group (both *p*’s < 0.01).

**Table 1:** Mean scores (standard deviations) on various questionnaires for children in the three groups

	SM group (*n* = 25)	Clinical control group (*n* = 17)	Non-clinical group (*n* = 127)
SMQ Selective mutism	30.80 (9.14)_a_	14.88 (9.82)_b_	8.27 (8.93)_c_
CASP Social anxiety	19.56 (3.44)_a_	11.00 (4.36)_b_	9.05 (5.27)_b_
BIQ Behavioral inhibition	66.24 (12.47)_a_	51.94 (10.27)_b_	39.06 (14.56)_c_
ASQ Autism spectrum problems	61.62 (15.93)_a_	66.53 (12.11)_a_	50.89 (14.64)_b_

*Note*. SM = Selective Mutism, SMQ = Selective Mutism Questionnaire, CASP = Children’s Anxiety Scale – Parent version, BIQ = Behavioral Inhibition Questionnaire, ASQ = Autism Spectrum Questionnaire. Within-row means that do not share similar subscripts differ at *p* < .05

### Correlations Between SM and Social Anxiety, Behavioral Inhibition, and ASD Problems

Correlations were computed among various measures (Table 2). The results first of all revealed that there were positive and statistically significant correlations between the SMQ on the one hand and the CASP, BIQ, and ASQ on the other hand. This indicated that selective mutism symptoms in children were positively associated with social anxiety symptoms, behavioral inhibition, and autism spectrum problems. Further analyses revealed that positive correlations between the SMQ and the CASP/BIQ were not only found for the total sample but also for the separate samples of non-clinical children and clinically referred children (which combined the SM and clinical control groups). The positive correlation between SMQ and ASQ was found in the total sample and in the non-clinical sample but not in the sample of clinically referred children (see Table 2). In addition, it should be mentioned that the SMQ was more convincing correlated with the interaction/communication problems subscale of the ASQ than with the odd/deviant behaviors subscale (total sample: *r*’s being 0.62, *p* < .001 versus 0.27, *p* < .001, *Z* = 6.32, *p* < .001; non-clinical sample: *r*’s being 0.63, *p* < .001 versus 0.48, *p* < .001, *Z* = 3.00, *p* = .001). Finally, when computing the correlations for the SM group only, again CASP social anxiety (*r* = .64, *p* = .001) and BIQ behavioral inhibition (*r* = .48, *p* = .01) were found to be significant correlates of SM symptoms, whereas the correlation between ASQ and SMQ was non-significant (*r* = .12, *p* = .57).

**Table 2:** Correlations among various questionnaire computed for the total sample (*N* = 169) and for the non-clinical (*n* = 127) and clinical (*n* = 42, SM and clinical control children combined) groups separately (respectively left and right values between parentheses)

	(1)	(2)	(3)
(1) SMQ Selective mutism			
(2) CASP Social anxiety	0.72* (0.59*/0.67*)		
(3) BIQ Behavioral inhibition	0.72* (0.60*/0.60*)	0.88* (0.88*/0.72*)	
(4) ASQ Autism spectrum problems	0.49* (0.59*/-0.10)	0.50* (0.59*/-0.14)	0.57* (0.59*/0.10)

*Note*. SM = Selective Mutism, SMQ = Selective Mutism Questionnaire, CASP = Children’s Anxiety Scale – Parent version, BIQ = Behavioral Inhibition Questionnaire, ASQ = Autism Spectrum Questionnaire. *p < .001

**Fig. 1:**
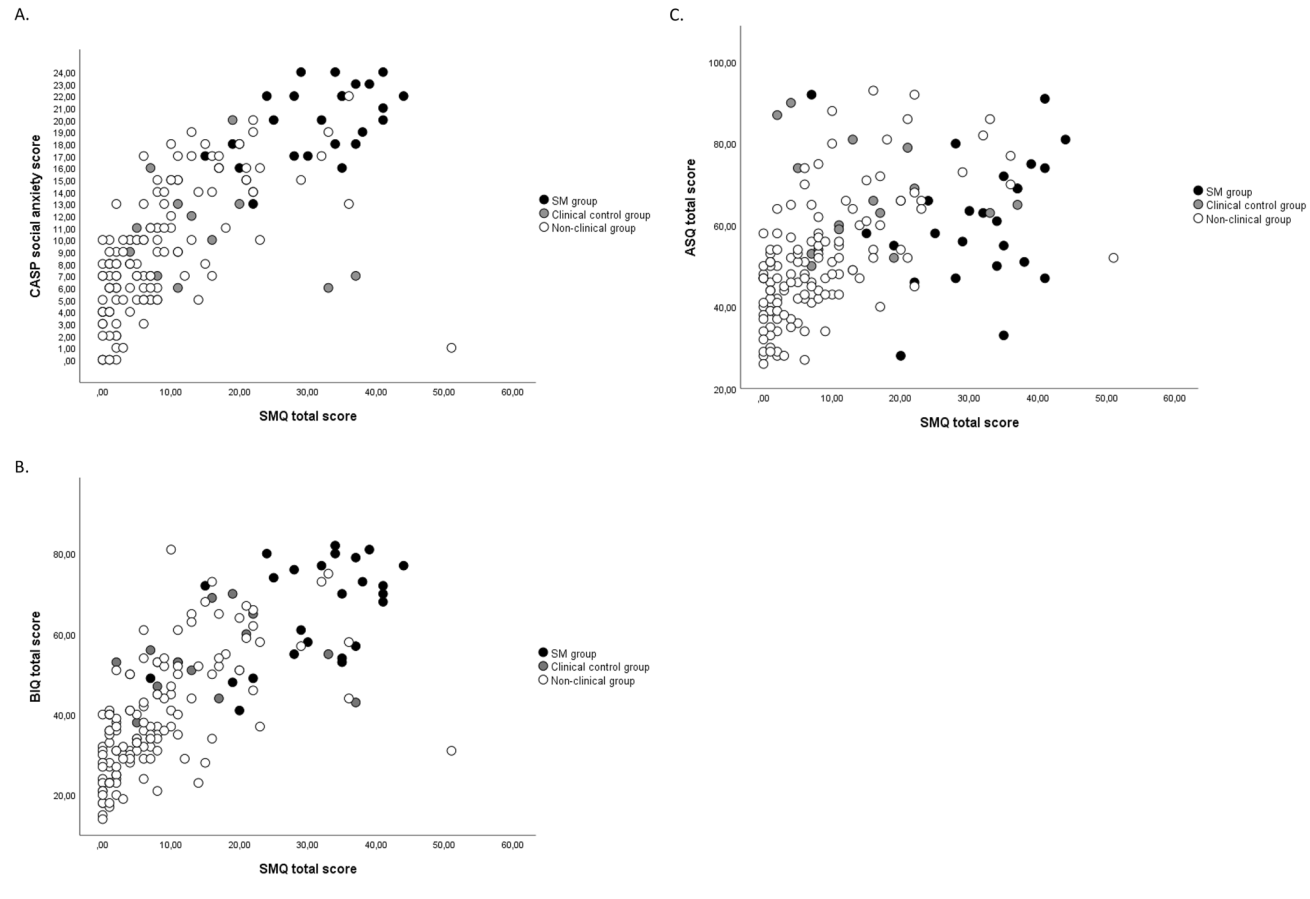
Scatterplots of the correlations (split by group membership) between SM and social anxiety (panel A), SM and behavioral inhibition (panel B), and SM and ASD symptoms (panel C). (Note: SM = Selective Mutism, SMQ = Selective Mutism Questionnaire, CASP = Children’s Anxiety Scale – Parent version, BIQ = Behavioral Inhibition Questionnaire, ASQ = Autism Spectrum Questionnaire)

To explore the relations among SM and the three other variables across the three groups in more detail, we inspected the scatterplots of these correlations (Fig. 1). For social anxiety and behavioral inhibition (panels A and B), the linear trends with symptoms of SM are clearly noticeable and it is also visible that children with SM are predominantly situated in upper right quadrant of the graph, which implies that many these children combine high levels of SM with high levels of social anxiety and behavioral inhibition. In the case of the relation between SM and ASD symptoms (panel C), the scatterplot is more diffuse: there are indeed some children combining high symptom levels of SM and ASD, but there are also quite a number of children with high levels of SM symptoms but lower levels of ASD and vice versa children with high levels of ASD but lower levels of SM.

### Unique Contributions to SM Symptoms

Stepwise linear regression analyses were carried out to examine the unique contributions of various symptom/temperament variables to symptoms of SM. In these analyses, CASP and ASQ (step 1), and BIQ (step 2) scores were entered into the equation as predictors of SMQ scores, which was the dependent variable. In the total sample, social anxiety and autism spectrum problems were both found to be significant predictors on step 1 (beta values being 0.64, *p* < .001 and 0.17, *p* < .01, respectively), together accounting for 54% of the variance in SMQ scores [*F*(2,166) = 98.92, *p* < .001]. When adding behavioral inhibition to the model on step 2, 2% of extra variance was explained [*F*(1,165) = 6.95, *p* < .01]. It was found that all three predictor variables made a unique and significant contribution to symptoms of SM: standardized beta values were 0.40 (*p* < .001) for CASP social anxiety, 0.30 (*p* < .01) for BIQ behavioral inhibition, and 0.12 (*p* = .05) for ASQ autism spectrum problems.

A separate regression analysis conducted in the non-clinical sample showed that both social anxiety (beta = 0.38, *p* < .001) and autism spectrum problems (beta = 0.37, *p* < .001) were again significant predictors on step 1, jointly explaining 44% of the variance in SM symptoms [*F*(2,124) = 48.87, *p* < .001]. When adding behavioral inhibition on step 2, only ASQ autism spectrum problems appeared to be a unique, significant predictor of SM symptoms (beta = 0.34, *p* < .001). A similar regression analysis performed in the clinical sample indicated that on step 1 only CASP social anxiety emerged as a significant predictor of SM symptoms (beta = 0.67, *p* < .001) [explained variance = 45%; *F*(3,38) = 11.65, *p* < .001] and this did not change after adding behavioral inhibition to the model on step 2 (beta = 0.46, *p* < .05).

Regression analyses that included the separate ASQ subscales instead of the total score revealed highly similar findings when the interaction/communication problems subscale was included in the model. The role of the odd/deviant behaviors subscale of the ASQ was rather inconsistent. In the total sample, this subscale did not explain unique variance in SM symptoms of the total sample. In the non-clinical sample, odd/deviant behaviors appeared to make a significant positive contribution to SM symptoms (step 1: beta = 0.24, *p* < .05; step 2: beta = 0.22, *p* < .05), but when analyzing the data of the clinical sample, this subscale was found to make a significant negative contribution (step 1: beta = − 0.26, *p* < .05; step 2: beta = − 0.29, *p* < .05).

### Proportion of SM Children Displaying Elevated/Clinical ASD Symptoms

**Fig. 2:**
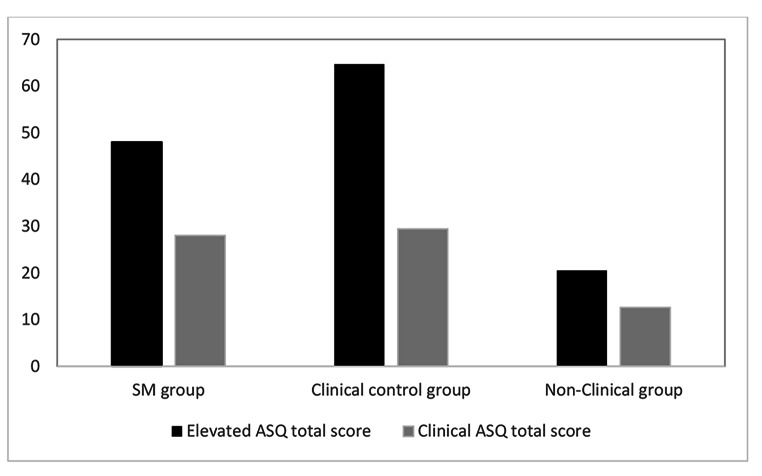
Percentage of children in each group for which parents reported elevated (cut-off score = 70, > percentile 90) and clinical (cut-off score = 89, > percentile 98) scores on the ASQ. (Note: SM = Selective Mutism, ASQ = Autism Spectrum Questionnaire)

Using normative data of the ASQ, we classified the proportions of children within each group who displayed elevated scores (i.e., total ASQ scores in the highest 10%) and clinical scores (i.e., total ASQ scores in the highest 2%). As can be seen in Fig. 2, elevated ASQ scores were noted in 48.0% of the children in the SM group, 64.7% of the children in the clinical control group, and 20.5% of the children in the non-clinical group. Fisher exact tests revealed no significant difference between the SM and clinical control groups (*p* = .35), while both groups were significantly different from the non-clinical group (*p*’s being 0.01 and < 0.001, respectively). The proportion of children displaying ASQ scores in the clinical range was more than twice as large in the SM and clinical control groups (28.0% and 29.4%) than in the non-clinical group (12.6%; see Fig. 2).

## Discussion

The purpose of the present study was to examine correlates of SM (symptoms) in 6- to 12-year-old non-clinical and clinically referred children, of whom some showed the prototypical signs of this condition. The results confirmed that social anxiety plays a prominent role in selective non-speaking behavior of children. More specifically, social anxiety was substantially and positively correlated with symptoms of SM, and this was true in the total sample, the separate samples of non-clinical and clinically referred children, and even within the small group of children with SM. This result is in keeping with similar previous correlational studies conducted in younger (3- to 6-year-old) children [14, 28] and clearly demonstrates that the frequency of selective non-speaking behavior is linearly associated with the severity of social anxiety symptoms. Furthermore, group comparisons indicated that children in the SM group were rated as displaying significantly higher levels of social anxiety symptoms as compared to children in the clinical control and non-clinical groups. This aligns well with the results of earlier investigations [8, 9, 44], which have also shown that children with SM display higher levels of social anxiety than non-clinical children and exhibit just as high levels of this type of anxiety symptoms as children with social anxiety disorder.

The link between SM and social anxiety has been further substantiated by a recent study of Vogel et al. [45] who conducted a network analysis of potential SM symptoms in a mixed sample of children and adolescents with and without an indication of SM (*N* = 899). These researchers noted that social anxiety symptoms seem to drive the SM symptom of ‘selectivity of speaking’, implying that social-evaluative fears to a large extent determine children’s muteness or taciturnity outdoors (while being talkative at home). All this evidence underlines the notion that social anxiety is an important feature of SM and that most children with this condition typically experience fear and anxiety in certain (challenging) social situations, which prompts them to refrain from speaking [6].

The results for behavioral inhibition were well in line with those found for social anxiety. That is, a clear and substantial correlation was found between symptoms of selective mutism and scores on the BIQ. In addition, children in the SM group displayed significantly higher levels of behavioral inhibition as compared to children in the clinical control and non-clinical groups. This confirms previous research that has also demonstrated that this temperamental trait plays a role in the selective non-speaking behavior, and in its extreme SM, of children [8, 13, 14]. In the meantime, it should be noted that most investigations (including the present study) on the relation between behavioral inhibition and SM are correlational in nature and hence one needs to be cautious with drawing conclusions in terms of cause-effect relations. After all, reticence in speaking in social situations has been generally viewed as a defining feature of an inhibited temperament [10, 46], and so one could argue that the link between behavioral inhibition and SM is to a certain extent tautological. Thus, we need more evidence showing that inhibition at an early age is predictive of (selective) non-speaking at a later point in children’s development [47].

Some support was also found for the link between SM and ASD symptoms. That is, children in the SM group were rated by their parents as displaying equally high levels of ASD symptoms as children in the clinical control group of whom a substantial proportion already had been officially diagnosed with this neurodevelopmental disorder. Furthermore, almost half of the children with SM (48.0%) displayed elevated scores on the ASQ (i.e., scores in the highest 10%) and 28.0% even exhibited scores above the clinical cut-off (i.e., scores in the highest 2%), which were percentages that were not significantly different from those noted in the clinical control group [25]. SM and ASD symptoms were only significantly correlated in the total sample and in the non-clinical group, whereas in clinically referred children and the group of children with SM in particular, no significant correlation between SM and ASD could be noted. Inspection of the scatterplot revealed a rather diffuse picture: there were indeed some children who combined high symptom levels of SM and ASD, but there were also quite a number of children with high levels of SM symptoms but lower levels of ASD and vice versa children with high levels of ASD but lower levels of SM.

The latter finding somehow makes sense when one takes into account that SM and ASD are both heterogeneous conditions. For example, latent profile analysis of children with SM has indicated that while (social) anxiety appears to be a common feature shared by most children with this condition, there seem to be different subgroups in which the selective non-speaking is accompanied by other characteristics, such as oppositional behavior and language problems [18, 19]. The possible co-occurrence of SM and ASD has long been discarded, but evidence is accumulating that there is a subgroup of children with this condition for whom the selective muteness is fueled by autistic symptomatology [27]. In a similar vein, ASD itself is also a complex and diversified psychiatric condition [48], which may manifest itself socially in various ways. For instance, in their seminal paper, Wing and Gould [49] already noted that some children with ASD can be defined as ‘aloof’, implying that they tend to withdraw themselves and often remain mute in social settings, whereas other children with ASD are socially ‘active (but odd)’, which means that they eagerly engage in interactions with others but in a less sensitive and tuned way. This is reminiscent of the notion that the interaction/communication problems and nonsocial impairments not always co-occur or even might be inversely related, particularly in higher-functioning samples of young people with ASD [50] and this may also account for the non-anticipated finding of the present study that the odd/deviant behavior problems were to some extent negatively associated with SM symptoms in the sample of clinically referred children.

However it may be, our results regarding the link between ASD and SM should be interpreted with caution. Additional analyses revealed that especially the interactive/communication problems subscale of the ASQ was responsible for the observed relationship with SM symptoms, and we cannot rule out the possibility that this relation reflected shared method variance. Obviously, the method used in this study (i.e., parent-rating scales) does not allow us to examine phenomenological differences in the selective non-speaking of children with SM and children with ASD. Therefore, it would be better to adopt a more experimental approach which could elucidate in what specific circumstances the communication impairments of these children occur: that is, children with ‘pure’ SM might show the non-speaking behavior particularly in anxiety-provoking situations, whereas children with ‘pure’ ASD might display the non-speaking behavior regardless of fear level. Obviously, such a test goes beyond the fact that ‘pure’ psychiatric conditions in clinical practice rarely exist (e.g., a substantial proportion of the children with ASD will also display fear and anxiety symptoms) [51] but could still provide valuable insights in the driving forces of the phenomenon of selective non-speaking in young people. Another viable method could be ecological momentary assessment: by repeatedly collecting data on children’s emotions, thoughts, and behavior in daily life, we could gain more knowledge on the mechanisms underlying selective non-speaking (e.g., fear and anxiety versus social skill deficits/lack of social motivation/social cognitive impairments typically seen in ASD) [45].

Altogether, it can be concluded that social anxiety, behavioral inhibition, and to a lesser extent autism spectrum problems are all three correlates of SM symptoms in middle childhood. The regression analyses, which enabled us to examine the unique contributions of social anxiety and ASD symptoms (step 1) and the temperament trait of behavioral inhibition (step 2) to symptoms of SM, yielded an inconsistent picture with expected as well as unexpected results. As anticipated, when exploring the relative contributions of social anxiety and ASD symptoms (step 1), it was found that social anxiety consistently emerged as a significant predictor. More precisely, in the total sample as well as in the separate samples of non-clinical and clinically referred children, CASP social anxiety accounted for a significant proportion of the variance in SM symptoms scores. The unique contribution of ASD symptoms to SM symptomatology was only statistically significant in the total sample and in the sample of non-clinical children. In the sample of clinically referred children, ASD symptoms did not explain a significant proportion of the variance, which could be explained by the aforementioned fact that this sample included ASD children with and without the prototypical signs of SM. An unexpected finding was that when entering behavioral inhibition in the regression models on step 2, this temperament trait only made a significant contribution to symptoms of SM when using the data of the total sample. Furthermore, in the non-clinical sample, we could not replicate our previous finding that the contributions of social anxiety and ASD symptoms would become non-significant once behavioral inhibition was added to the model [28]. In fact, it was found that ASD problems emerged as the only unique predictor of SM symptoms in these typically developing children. It is difficult to explain these inconsistent findings, but one possibility could be that SM symptoms in non-clinical preschool children represent a more normative phenomenon (i.e., reflect an avoidant coping strategy that is best explained by an inhibited temperament), whereas in older children selective non-speaking is more pathological and unusual in nature (and thus better explained by autism-spectrum features).

The findings of this study have to be seen in light of a number of limitations. First of all, no standardized clinical interviews were used to validate the diagnoses of the children in the clinical sample. Note however that the outpatient treatment center – where the study was conducted – relied on a Longitudinal Expert All Data (LEAD) approach [52] to establish the diagnoses of the children, and this method has been demonstrated to possess considerable validity [53, 54]. Second and related to this point, although the children in the SM group were all displaying the prototypical selective non-speaking behavior, 40.0% had not yet officially been given the diagnosis of SM because the clinicians were still deliberating about their exact classification. In many of these cases, the classification of ASD or social anxiety disorder was considered, which illustrates clinicians’ struggles with the dimensional nature of psychopathology (i.e., the severity of SM may differ across children and so ‘lighter’ cases of SM might be seen as social anxiety) and the issue of mutual exclusivity versus co-occurrence of diagnostic categories (i.e., the differential diagnosis versus comorbidity of SM and ASD) [55]. Third, the number of children included in the SM and clinical control groups were relatively small, which may have undermined the statistical power in some analyses. Fourth, some questions can be raised regarding the ‘normality’ of the non-clinical group. For example, according to normative data of the ASQ, a considerable percentage of these non-clinical children displayed elevated scores (i.e., 20.5%), with 12.6% even exhibiting ASD symptomatology in the clinical range. In addition, although none of these children was currently receiving clinical care, 11% of them had previously been diagnosed with some type of psychopathology. It is possible that the recruitment at school by means of a flyer with the title “Children who sometimes do not speak” attracted a disproportionate number of parents of children with (neuro)developmental problems. Fifth and finally, to enable comparison with previously collected data of younger children, we decided to use the preschool version of the CASP in this population of 6- to 12-year-olds. While the scores on this measure showed sufficient to good reliability coefficients, the validity has of course not been tested in this age group. Nevertheless, the use of the preschool version of the CASP can be justified because its items are also relevant for older (primary school) children as they typically reflect the fear of being negatively evaluated in social situations, which is the key feature of social anxiety disorder [1].

## Summary

The present study explored psychopathological and temperamental correlates of SM in a mixed sample of clinically referred and non-clinical children. The results underline the relation between SM and anxiety pathology: that is, symptoms of SM were clearly associated with social anxiety symptoms as well as with the temperament trait of behavioral inhibition, which has been shown to be an important developmental precursor of (social) anxiety disorder(s) [11, 12]. Clearly, this justifies the current classification of SM as an anxiety disorder [4, 5]. In the meantime, the results of the current investigation also suggest that autism spectrum problems play a role in SM and that there appears to be a subgroup of children who display selective non-speaking behavior within the context of ASD [27]. This supports the notion of some scholars [21] who have argued that SM is a rather heterogeneous psychiatric condition that besides anxiety pathology may also have (neuro)developmental antecedents including ASD and language disorders. Clearly, this also has implications for clinical practice: psychologists and psychiatrists who encounter a child with SM should not merely focus on fear and anxiety symptoms but also have an eye for (neuro)developmental problems including ASD. It is an improvement that the latest edition of the DSM allows the comorbidity of SM and ASD [56] and so a proper assessment of anxiety as well as developmental pathology may guide clinicians to a better treatment indication. In case the selective non-speaking behavior is purely anxiety-based a cognitive-behavioral approach seems most appropriate, but in case the SM is also fueled by ASD- or language-related problems, other interventions (e.g., social skills training, speech and language therapy) are also indicated [2].

## Data Availability

Data and materials used for this study can be obtained from the first author.
